# Unveiling the Impact of Peanut Consumption on Telomere Length in Young and Healthy Individuals: Insights from the ARISTOTLE Study: A Randomized Clinical Trial

**DOI:** 10.3390/antiox14040467

**Published:** 2025-04-14

**Authors:** Daniel Torres-Oteros, Isabella Parilli-Moser, Emily P. Laveriano Santos, Nerea Becerra-Tomás, Héctor Sanz-Lamora, Sara Hurtado-Barroso, Diego Haro, Pedro F. Marrero, Rosa M. Lamuela-Raventos, Joana Relat, Silvia Canudas

**Affiliations:** 1 Department of Nutrition, Food Sciences and Gastronomy, School of Pharmacy and Food Sciences, Food Torribera Campus, University of Barcelona, 08921 Santa Coloma de Gramenet, Spain; d.torres.oteros@ub.edu (D.T.-O.); h.sanz.lamora@ub.edu (H.S.-L.); dharo@ub.edu (D.H.); pedromarrero@ub.edu (P.F.M.); lamuela@ub.edu (R.M.L.-R.); 2 Maria de Maeztu Unit of Excellence, Institute of Nutrition and Food Safety, University of Barcelona (INSA-UB), 08921 Santa Coloma de Gramenet, Spain; 3 CIBER Physiopathology of Obesity and Nutrition (CIBEROBN), Instituto de Salud Carlos III, 28029 Madrid, Spain; isaparillim@gmail.com (I.P.-M.); emily.laveriano@ub.edu (E.P.L.S.); nerea.becerra@urv.cat (N.B.-T.); sara17hb@gmail.com (S.H.-B.); 4 Institut de Salut Global (ISGlobal), 08003 Barcelona, Spain; 5 Universitat Rovira i Virgili, Department of Basic Medical Sciences, Unitat de Salut Pública i Epidemiologia Nutricional, Nutrition and Mental Health (NUTRISAM) Research Group, 43201 Reus, Spain; 6 Institut d’Investigació Sanitària Pere Virgili (IISPV), 43003 Tarragona, Spain; 7 Department of Biomedical Sciences, Universitat Internacional de Catalunya, 0801733 Barcelona, Spain; 8 Institute of Biomedicine of the University of Barcelona (IBUB), 08028 Barcelona, Spain

**Keywords:** peanut, telomere length, MUFA, phenolic compounds, bioactive compounds, coumaric acid

## Abstract

Diet is a potential modulator of telomere length (TL), but the impact of individual dietary components, such as nuts, on TL in young, healthy individuals remains underexplored. Peanuts are rich in bioactive compounds that may influence TL. Therefore, to fill this gap of knowledge, this study aimed to investigate the effect of peanut consumption on TL in this specific population. Fifty-eight young, healthy individuals were randomized to one of three different intervention groups for 6 months each: (1) 25 g/day of skin-roasted peanuts (SRP); (2) 32 g/day of peanut butter (PB); (3) 32 g/day of a control butter (CB) (based on peanut oil). TL was measured by quantitative real-time PCR in saliva at baseline and at the end of the intervention. Our findings revealed significant between-group differences in TL changes, particularly between the SRP and CB groups over 6 months (mean difference: 0.53; 95% CI: 0.01, 1.05; *p*-value = 0.048). No significant difference was observed between PB and CB groups (mean difference: 0.12; 95% CI: –0.42, 0.66; *p*-value = 0.66). This study provides novel insights into the impact of peanut consumption on TL maintenance in young and healthy individuals. The findings highlight the potential benefits of incorporating peanuts into the diet as a means of promoting cellular health and longevity. Further research is warranted to elucidate the underlying mechanisms and validate these findings across diverse populations and longer time frames.

## 1. Introduction

Peanuts (*Arachis hypogaea*) are a leguminous plant from South America that was introduced in Europe in the 18th century [1]. Although peanuts belong to the same family as legumes or beans, they are typically included in the group of nuts due to their similar nutritional composition [1]. Peanuts have a great nutritional profile, being rich in unsaturated fatty acids mainly, oleic, and linoleic acids, dietary fiber, B and E vitamins, potassium, phytonutrients such as (poly)phenols [2], and other bioactive compounds of potential interest for human health. These (poly)phenols, including flavonoids and stilbenes like resveratrol, exhibit potent antioxidant properties, counteracting oxidative stress by scavenging free radicals and reducing lipid peroxidation [3]. Extensive research supports that regular peanut consumption is associated with reduced risk of cardiovascular disease incidence and mortality [4], diabetes [5], certain types of cancer, and overall mortality [6,7]. Interestingly, these age-related conditions have been associated with shortened telomere length (TL) [8,9]. Within the ARISTOTLE trial, performed in young and healthy individual, Parrilli-Moser et al. described the improvement in vascular cognitive performance with the daily consumption of this peanuts [10,11].

Telomeres, comprised of repetitive sequences (TTAGGG), play a vital role in maintaining chromosome stability and integrity [12]. However, telomeres shorten with each cell division and their progressive attrition is linked to replicative senescence, a hallmark of aging [13]. While population-based studies revealed a decrease in TL with age [14], the rate of attrition is variable across individuals and life stages [15]. The length of telomeres in individuals is a dynamic process influenced by both initial TL at birth and the rate of erosion throughout life [16]. During infancy and childhood, TL shortens rapidly due to high cellular turnover [17,18], but the rate stabilizes to about 50–60 base pairs per year in adulthood. In older age, telomere shortening accelerates, with an estimated loss of 5–6 kilobases in individuals aged over 60 years [19]. This intricate process underscores the importance of analysing TL in healthy young individuals, as it not only provides insights into early aging markers but also helps identifying potential risk factors for age-related diseases, offering valuable opportunities for disease prevention.

TL dynamics are intricately influenced by various modifiable lifestyle factors such as physical activity, obesity, stress, or diet [20]. A growing body of research has shown that healthy diets rich in anti-inflammatory and antioxidant components could delay TL shortening [21]. Indeed, oxidative stress accelerates telomere shortening by increasing cellular DNA damage. In this context, antioxidants play a crucial role counteracting oxidative stress and protecting cells from damage. Peanuts, in particular, are rich in bioactive compounds such as resveratrol, flavonoids, and phytosterols, which have potent antioxidant and anti-inflammatory properties. Although studies specifically investigating “peanut and telomere” are scarce, the high content of these bioactive compounds in peanuts justifies further exploration of their impact on TL.

While epidemiological studies on TL in adults have provided valuable insights, there is a growing interest in investigating TL during early life stages due to its potential connection with adverse outcomes in later adulthood [22,23]. However, research on the effects of nut consumption on telomeres in young and healthy individuals remains limited [24], with no studies specifically addressing peanut consumption.

Therefore, building on data from the ARISTOTLE study (NCT04324749), a randomized controlled trial involving healthy young participants aged 18 to 33 years, this study aimed to explore the impact of daily consumption of skin-roasted peanuts (SRP) and peanut butter (PB) in preventing telomere shortening in young, healthy individuals.

## 2. Materials and Methods

### 2.1. Study Population and Study Design

**Figure A1:**
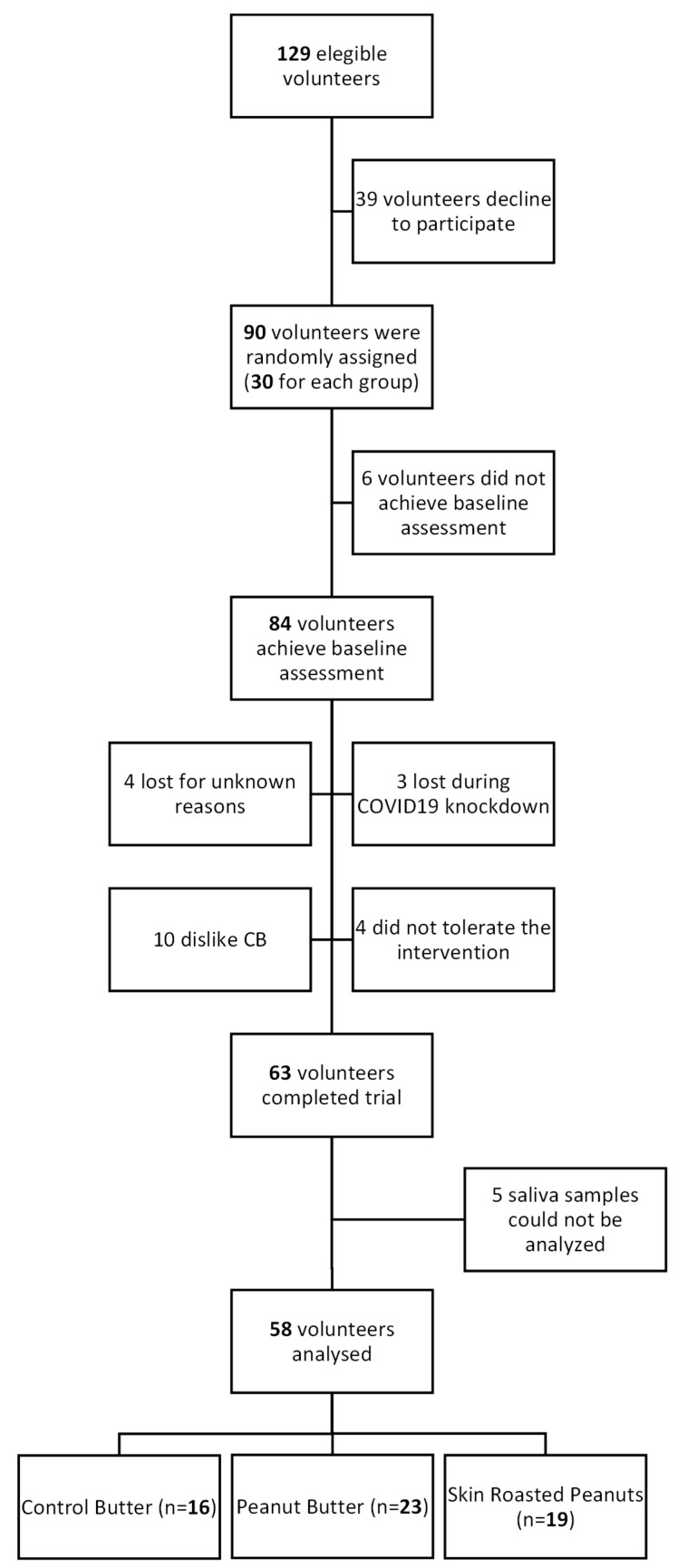
Flowchart of ARISTOTLE study.

The present study was carried out within the frame the ARISTOTLE study (NCT04324749), a three-arm parallel randomized controlled trial conducted in 2019–2020 in sixty-three healthy young adults (18–33 years) from the Food and Nutrition Torribera Campus of the University of Barcelona and surroundings, with the main objective to assess the impact of daily SRP and PB intake on the organism, evaluating their prebiotic and postbiotic effects. Exclusion criteria included a history of chronic diseases (e.g., cardiovascular diseases, cancer, diabetes), peanut allergy or intolerance, body mass index (BMI) over 25 kg/m^2^, active smoking, high alcohol consumption and other toxic habits. More details of the study design have been previously published [11]. In the present sub-study, five participants were excluded from the total sample who completed the study, due to incomplete data of telomere length. Finally, fifty-eight participants were included (Figure A1). The study protocol was approved by the Bioethics Commission of the University of Barcelona (Institutional Review Board: IRB 00003099) and carried out following the Declaration of Helsinki. Participants provided written informed consent prior to the start of the trial.

### 2.2. Intervention

After a two-week peanut-free run-in period, participants were randomly assigned to one of three 6-month duration interventions: 25 g/day of SRP or 2 tablespoons (32 g)/day of PB or 2 tablespoons (32 g)/day of a control butter (CB). The CB was made with peanut oil, free of phenolic compounds and fiber. Different amounts of peanuts and peanut butter were selected based on guidelines from official nutrition organizations, which define a standard portion size as 2 tablespoons (32 g) for peanut butter and 25–30 g for peanuts. Both peanuts (1 g of salt/100 g) and peanut butter (0.84 g of salt/100 g) contain salt as one of their ingredients. Participants maintained their usual dietary habits and consumed the assigned product at their convenience throughout the day. However, consumption of wine, grapes, dark chocolate (>70%), and berries was restricted due to their high resveratrol content, a phenolic compound which is also present in peanuts. Additionally, nuts (pistachio, walnuts, almonds, hazelnuts) were excluded from the diet due to their similar nutritional content to peanuts (see Parilli-Moser et al., 2021) [11].

### 2.3. Anthropometric, Biochemical, and Clinical Measurements

The following measurements were taken with the participants in fasting conditions at the beginning and end of the trial. Body weight and composition (body fat and muscle percentage) were assessed using a tetrapolar OMRON BF511 electronic scale, with participants wearing light clothes and no footwear. Height was measured in the standing position using a portable stadiometer. BMI was calculated as weight divided by height squared (kg/m^2^). Waist circumference was measured with a Holtain tape measure positioned at the midpoint between the lower margin of the last rib and the top of the iliac crest, while hip circumference was measured at the level of the upper trochanters. Both measurements were utilized to compute the waist-to-hip ratio. Blood pressure was recorded three times at two/three-minute intervals using an OMRON M6 digital monitor, with volunteers seated. Blood glucose and lipid parameters, including total cholesterol, high-density lipoprotein cholesterol (HDL-c), low-density lipoprotein cholesterol (LDL-c), and triglycerides (TG), were analysed at an external laboratory (Cerba internacional, Barcelona, Spain) employing enzymatic methodologies.

### 2.4. Dietary, Physical Activity, and Sociodemographic Variables

Covariate data were collected by professional staff members using general questionnaires about socio-demographic variables (i.e., sex, age, level of education), dietary intake and physical activity at baseline and at the end of the intervention. The diet was evaluated using a validated 151-item semi-quantitative food frequency questionnaire to quantify the usual food intake, estimated according to Spanish food composition tables [25]. Physical activity was measured as the metabolic equivalent of task-minutes per week (MET/week) using the Spanish validated version of the Minnesota Leisure-Time Physical Activity Questionnaire [26,27].

### 2.5. Telomere Length Assessment

Genomic DNA was isolated from frozen saliva samples (collected at 0 and 6 months) using lysis buffer and isopropanol extraction following the manufacturer’s instructions. This extraction method is vastly used in epidemiological studies [28]. TL can vary across different cells or tissues; however, salivary TL has been correlated with TL measured in whole blood [29], buffy coat [30], and leukocytes [31]. In this study, TL was measured by real-time PCR (qPCR) [32], using the SYBR Select Master Mix for CFX (Applied Biosystems, Waltham, MA, USA). The protocol was adapted from Nathan J O’Callaghan [33] and we used the following primers and standards: TeloF (CGGTTTGTTTGGGTTTGGGTTTGGGTTTGGGTTTGGGTT), TeloR (GGCTTGCCTTACCCTTACCCTTACCCTTACCCTTACCCT), *36b4*F (CAGCAAGTGGGAAGGTGTAATCC), *36b4*R (CCCATTCTATCATCAACGGGTACAA), Telomere standard (14 tandem copies of TTAGGG), and *36b4* standard (CAGCAAGTGGGAAGGTGTAATCCGTCTCCACAGACAAGGCCAGGACTCGTTTGTACCCGTTGATGATAGAATGGG). This approach employs *36b4* single-copy gene as a reference for each sample. The quantification of the relative copy numbers of telomeres (T) and a single copy gene (*36b4*; S) was performed in triplicates using a Bio-Rad CFX96 thermocycler with samples collected before and after the intervention. TL is expressed as a ratio of these two parameters (T/S ratio). A calibration curve with standards of Telomere (1.8 × 10^5^ to 1.8 kb) and *36b4* (2.63 × 10^5^ to 2.63 diploid copies) in 10-fold dilutions with a linearity agreement of R^2^ > 0.99 were included in each 96-well plate.

### 2.6. Statistical Anaysis

The normality of distribution was analysed by the Shapiro–Wilk test. Most of the variables did not follow a normal distribution, then differences in baseline characteristics between groups were assessed using the Kruskal–Wallis test for continuous variables, followed by the post hoc Dunn’s multiple comparisons test when significant differences were observed. The chi-square test was used to compare proportions among categorical variables. Means and standard deviations (SD) or numbers and percentages, as appropriate, are shown for the description of baseline characteristics according to the intervention group.

The primary outcome was TL change over 6-months by intervention group. Additional analyses included 6-month changes in anthropometric, biochemical, clinical, and dietary variables. The generalized estimating equation (GEE) model for repeated measurements (with identity link function, first-order autoregressive correlation, and robust standard error parameters) was used to estimate the effect of the interventions on the aforementioned variables. Two adjustment models were generated to avoid other factors influencing the outcomes. Model 1 was adjusted for sex and age. Model 2 was further adjusted for BMI (kg/m^2^), physical activity, and total energy intake (kcal/day). Data are expressed as adjusted mean differences and their 95% confidence intervals (CI). The proportion of participants in each group achieving an accelerated telomere shortening (ΔTL ≤ percentile 20) was also estimated.

In addition, linear regression models were used to evaluate associations between changes in anthropometric, biochemical, and dietary variables (which showed statistically significant differences between groups after 6 months of intervention) and changes in telomere length. Three adjustment models were used: Model 1 was adjusted for sex and age; model 2 was adjusted as for model 1 plus BMI (kg/m^2^), physical activity, and total energy intake (kcal/day); and model 3 was adjusted as for model 2 plus telomere length at baseline.

Due to the non-normality of most of the variables, their changes (difference between 6-months and baseline) were normalized and scaled in multiples of 1 SD using the Blom inverse normal transformation to perform this analysis [34].

All statistical analyses were performed with Stata software, version 16.0 (Stata Corp LP, College Station, TX, USA). Significance testing was considered for *p*-value < 0.05.

## 3. Results

### 3.1. Baseline Characteristics of Participants

A total of 58 participants from the ARISTOTLE study (mean age 22.74 ± 3.24 years; BMI was 22.45 ± 3.02 kg/m^2^, indicating young and healthy body weight), were included. The baseline characteristics (before the intervention) of participants by intervention group are presented in Table 1. No significant differences were observed among the study groups, except for plasmatic HDL-c (*p*-value = 0.016) and *m*-coumaric acid intake (*p*-value = 0.035).

### 3.2. Effect of the Intervention on Telomere Length

**Figure 1:**
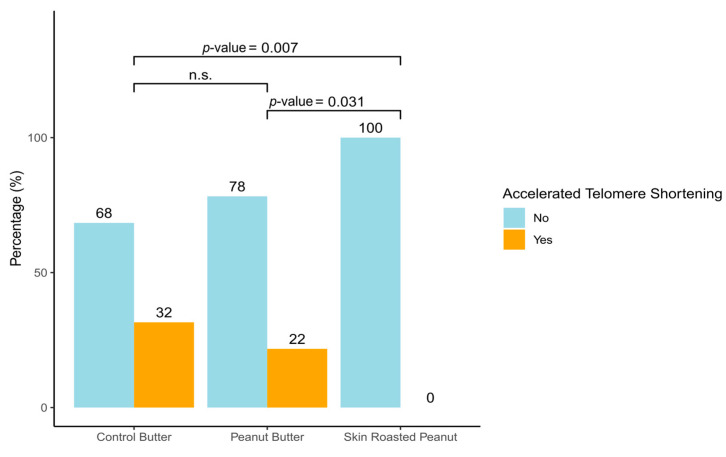
Accelerated telomere shortening by intervention group. Accelerated telomere shortening was assessed by monitoring change in telomere length over a 6-month follow-up period, specifically targeting a threshold of percentile 20 or lower (ΔTL ≤ percentile 20; ΔTL ≤ p20).

The changes in TL after 6 months of the SRP or PB interventions compared with the CB intervention are shown in Table 2. The SRP group showed a significant increase in TL over time compared to the CB group (adjusted mean difference: 0.53; 95% CI: 0.01, 1.05; *p*-value = 0.048), after adjusting for sex, age, BMI, physical activity, and energy intake. No significant between-group differences in TL change from baseline were observed between the PB group and the CB group. Additionally, we evaluated the percentage of participants achieving an accelerated telomere shortening (defined as <20th percentile) and found that the participants consuming SRP were the only group not exhibiting accelerated telomere shortening. In contrast, 22% of those in the PB group and 38% in the CB group showed greater rates of telomere shortening (Figure 1).

### 3.3. Relationships Between Variables That Changed with the Intervention and Telomere Length

The between-group differences (SPR vs. CB, PB vs. CB) in anthropometric, biochemical, and clinical parameters over 6 months, analysed using the aforementioned models, are presented in Table A1. Regarding anthropometric measures, there were significant between-group differences (SRP vs. CB) in waist-to-hip ratio (adjusted mean difference: −0.03; 95%CI: −0.05, −0.01; *p*-value = 0.027), but no differences were observed between interventions in other related parameters such as body fat (%) or visceral fat. In terms of biochemical parameters, the SRP group exhibited a significantly greater increase in HDL cholesterol levels with the intervention (adjusted mean difference: 0.20; 95%CI: 0.02, 0.37; *p*-value = 0.030). It is worth noting that the SRP group already had higher baseline HDL cholesterol levels than the CB group at the beginning of the study.

Additionally, we examined between-group differences in changes in dietary intake over 6 months of intervention (Table A2). The SRP group showed a higher increase in the intake of sugars (adjusted mean difference: 11.59; 95%CI: 0.04, 23.15; *p*-value = 0.049) and fiber (adjusted mean difference: 4.88; 95%CI: 1.77, 7.99; *p*-value = 0.002) compared with the CB group. Fiber intake was also higher in the PB group (adjusted mean difference: 3.65; 95%CI: 0.45, 6.85; *p*-value = 0.025) than the CB group. Regarding micronutrients and phytochemicals, both SRP and PB groups had a higher intake of vitamin E (adjusted mean difference: 2.76; 95%CI: 0.85, 4.67; *p*-value = 0.005 and adjusted mean difference: 3.56; 95%CI: 1.25, 5.84; *p*-value = 0.002, respectively) than the CB group. Furthermore, the intake of phytochemicals specific to peanuts, such as coumaric acids and resveratrol, was notably higher in both intervention groups compared to the CB group, However, *o*-coumaric acid had a higher intake only in the SRP group (adjusted mean difference: 1.45; 95%CI: 0.85, 2.04; *p*-value < 0.001), as has been observed previously in this trial by Parilli-Moser et al. [11].

Following the observed modifications in anthropometric and biochemical variables throughout the intervention, linear regressions were carried out with TL as dependent variables, using three different models. Table 3 shows the linear regressions using the most adjusted model, while the rest are shown in Table A3. A positive association between monounsaturated fatty acids (MUFA) intake and TL was identified when adjusting for sex and age (β: 0.58; 95%CI: 0.11, 1.05; *p*-value = 0.016), This association remained significant after further adjustments for BMI, physical activity, and energy intake (β: 0.46; 95%CI: (0.02, 0.93; *p*-value = 0.048), and persisted when baseline TL was included in the model (β: 0.35 (0.01, 0.69), *p*-value = 0.046).

Regarding specific phytochemicals, resveratrol and anthocyanins showed an initial positive association with TL (β: 1.52; 95%CI: 0.37, 2.67; *p* = 0.011 and β: 0.79; 95%CI: 0.02, 1.56; *p*-value = 0.044, respectively). However, these associations were no longer significant after adjusting for baseline TL (model 3). On the other hand, *m*-coumaric acid exhibited a significant positive association with TL only in model 3 (β: 0.42; 95%CI: 0.18, 0.67; *p*-value = 0.012).

## 4. Discussion

In the present sub-study, we have evaluated the impact of peanut consumption (SRP and PB), on TL in young, healthy individuals from the ARISTOTLE study. Our results revealed a positive impact of SRP on TL, whereas no significant differences were observed between the PB and CB groups (Table 2). Moreover, only SRP reveal a protective role against accelerated telomere shortening (Figure 1). While extensive research has explored dietary influences on TL, studies focusing on young, healthy populations remain scarce. To the best of our knowledge, this is the first randomized controlled trial investigating the specific impact of peanut consumption on TL in this demographic.

Telomere attrition, primarily driven by oxidative stress and inflammation, is a well-documented process [35]. Given that edible plants are rich in bioactive compounds with antioxidant and anti-inflammatory properties, there is a strong rationale to suggest that regular consumption of such foods, including peanuts, could help mitigate telomere shortening [36]. Recent systematic reviews have highlighted the positive association between TL and antioxidant-rich diets, emphasizing specific nutrients [21], increased fruits and vegetables intake [21], and adherence to plant-rich dietary patterns [36] such as the Mediterranean diet [21,37]. In this context, nut consumption has been associated with longer telomeres [38], suggesting a potential role delaying cellular aging and senescence.

Few randomized controlled trials [39,40,41,42] have examined the impact of nut consumption on TL across diverse populations. Consistent with our findings on SRP, previous studies have reported promising effects of walnuts consumption in elderly individuals [39] and pistachio intake in prediabetic individuals [40]. Specifically, walnuts have been associated with prevention of telomere shortening, and pistachios intake has been linked to increased expression of telomerase-related genes [39,40]. Moreover, the PREDIMED-Plus Study reported an increase in TL over time both groups following a Mediterranean diet, regardless of energy restriction and exercise intervention [41]. Notably, nut consumption significantly increased in both groups, which may have contributed to maintain TL. However, the PREDIMED study, conducted in adults at high cardiovascular risk, found that mixed nut supplementation (walnuts, hazelnuts, and almonds) accelerated telomere shortening compared to a low-fat control diet [42]. Discrepancies in results may stem from differences in participant characteristic, TL assessment methodologies, and the types of nuts consumed.

Our study focuses on young, healthy adults, suggesting that the effects of nut intake on TL might vary based on baseline health status. However, it is plausible that SRP could also exert a protective effect in elder population. Nevertheless, when transferring this study to older individuals, careful consideration should be given to salt intake from peanuts. Consuming unsalted peanuts would be advisable. Additionally, the distinct nutritional profile of peanuts compared to the mixed nuts in PREDIMED study could differentially influence telomere biology [43].

It is noteworthy that, while SRP consumption showed a beneficial effect on TL, no significant differences in TL changes were observed between the PB and CB groups. Although commercially available peanut butter often contains added fats, the peanut butter used in our study was composed solely of peanuts and salt. The key distinction between whole peanuts and peanut butter lies in their processing methods, such as grinding and homogenization. These processes modify the fiber matrix and may enhance the bioavailability of polyphenols, as suggested by findings from the ARISTOTLE study [11]. In contrast, whole peanuts, require grater digestive effort, particularly involving the gut microbiota, which may lead to increased production of short-chain fatty acids (SCFAs) [44]. Notably, our previous research identified elevated fecal SCFAs levels exclusively in the SRP group, with a negative correlation between SCFAs and biomarkers of depression and cortisol, factors closely linked to aging and telomere attrition [45].

Additionally, we explored specific peanut-derived compounds that may contribute to telomere maintenance. Our findings suggest that dietary intake of MUFA and *m*-coumaric acid may exert protective effect on telomere integrity. Previous studies have shown that MUFA [46] and hydroxycinnamic acid derivatives [47] such *m*-coumaric acid positively influence lipid metabolism, exhibit antioxidant activity, and reduce oxidative stress mechanisms that have been linked to improved metabolic health. These bioactive compounds are abundant in health-promoting dietary patterns like the Mediterranean diet, which has been widely associated with longevity and a reduced risk of chronic disease.

Despite these promising findings, previous studies have yielded inconsistent results regarding the effects of these compounds on TL. For instance, the Nurses’ Health Study reported an association between adherence to the Mediterranean diet and longer leukocyte telomeres; however, no significant correlations were observed with individual dietary components, including the MUFA-to-fatty-acid ratio were detected [48]. Similarly, research in older populations [49,50] has produced mixed findings, with some studies even noting negative correlations. This variability underscores the need for further research to determine how factors such as dietary sources, age, and lifestyle modulate the impact of these compounds on TL.

Regarding *m*-coumaric acid, no studies have directly examined its relationship with TL. However, its potent antioxidant and anti-inflammatory properties suggest a potential protective role in telomeres maintenance by mitigating oxidative stress and inflammation, two key drivers of telomere shortening [47]. Our findings highlight the need for further research to elucidate the role of *m*-coumaric acid in telomere biology.

More broadly, investigating the effects of these bioactive compounds, particularly their presence in peanuts and other plant-based foods, could provide valuable insights into their impact on TL regulation and cellular health. Given that nutrients influencing oxidative stress and inflammation are known to modulate telomere dynamics, nutrition represents a promising avenue for understanding aging and age-related diseases such as obesity, insulin resistance, and cardiovascular conditions, all of which are characterized by heightened inflammation and oxidative stress [51,52,53]. Future research should focus on elucidating the mechanisms underlying these effects and validating these findings in more diverse populations over extended study periods.

Our findings are particularly relevant, as telomere attrition, as previously mentioned, has been linked to metabolic dysfunction and increased risk of age-related diseases including cardiovascular diseases, diabetes, and cancer [54]. Peanut consumption may help mitigate these risks by reducing oxidative stress and inflammation, two key contributors to telomere shortening. Indeed, a recent meta-analysis reported that peanut intake was associated with a lower incidence of cardiovascular disease and mortality, as well as reduced rates of stroke and coronary heart disease [4]. Interestingly, no such associations were observed with peanut butter, possibly due to the addition of salt and fats as noted above. Moreover, another study found an inverse associated between peanut consumption and overall cancer risk [55].

One of the strengths of our study is the homogeneity of the participant pool, as all individuals were young (18–33 years) and recruited based on standardized criteria minimizing age-related confounding effects on TL. This reduces confounding bias related to age, a factor known to affect TL. Additionally, targeting a healthy population aligns with the goal of promoting preventive health strategies. However, this homogeneity also limits the generalizability of our findings to broader populations. Our study focused solely on a young population, where telomeres are inherently longer. It would be highly interesting to investigate the effects of peanut consumption in an older population, where telomere attrition is more pronounced.

Another potential limitation is the small sample size for each group. The ARISTOTLE trail was initially designed to achieve 80% of statistical power; however, due to a higher-than-expected dropout rate, this was reduced to 60%. Therefore, larger studies are needed to confirm these findings. Moreover, the participant self-selection is another limitation of the study because individuals who enrolled in the intervention may have been more health-conscious than the general population, potentially affecting the representativeness of the results. Furthermore, the absence of a peanut-free control group is a notable limitation, as the control group consumed a paste made from peanut oil, which may have introduced confounding effects.

## 5. Conclusions

In conclusion, this study underscores the potential benefits of peanut consumption, particularly whole peanuts (SRP), in supporting telomere maintenance and slowing cellular aging in young, healthy individuals. Rich in MUFA and *m*-coumaric acid, peanuts may contribute to healthy aging and lower risk of age-related diseases.

The observed differences between SRP and peanut butter (PB) highlight the impact of processing methods on fiber structure, nutrient bioavailability, and SCFA production, which may partially explain the distinct effects of both interventions. However, further research is needed to validate these findings in larger and more diverse populations over extended study periods, with a particular focus on elder populations. Moreover, it would be interesting to investigate the molecular mechanisms underlaying the effects of peanuts in a future mouse model study. Expanding this line of research could provide valuable insights into the role of dietary interventions in promoting longevity and preventing chronic diseases.

## Figures and Tables

**Table 1:** General characteristics of study population at baseline by intervention group.

	SRP (*n* = 19)	PB (*n* = 23)	CB (*n* = 16)	*p*-Value
**Female, *n* (%)**	12 (66)	18 (78)	10 (63)	0.528
**Age (years)**	22.42 ± 3.32	23.43 ± 2.90	22.38 ± 3.50	0.308
**Educational level, *n* (%)**				0.512
University students	10 (52.60)	11 (48)	11 (68.80)	
Graduated	9 (47.40)	12 (52)	5 (31.20)	
**Physical activity (MET/week**)	4935 ± 2191	4703 ± 2381	4920 ± 1670	0.769
**Anthropometric measurements**				
Weight (kg)	64.47 ± 10.68	60.10 ± 7.72	64.68 ± 10.68	0.228
BMI (kg/m^2^)	22.38 ± 3.61	22.19 ± 2.60	22.79 ± 2.86	0.793
Waist circumference (cm)	73.68 ± 8.12	71.28 ± 5.53	75.38 ± 6.30	0.156
Waist-to-hip ratio	0.74 ± 0.06	0.74 ± 0.05	0.77 ± 0.05	0.166
Body fat (%)	26.71 ± 8.48	28.45 ± 7.88	26.51 ± 7.41	0.634
**Blood Pressure**				
Systolic blood pressure	112 ± 7.33	110 ± 8.87	111 ± 10.20	0.327
Diastolic blood pressure	72.28 ± 7.93	72.87 ± 6.20	69.98 ± 9.37	0.476
**Biochemical parameters**				
Glucose (mmol/L)	4.58 ± 0.44	4.59 ± 0.35	4.44 ± 0.24	0.307
Triglycerides (mmol/L)	0.71 ± 0.21	0.85 ± 0.35	0.78 ± 0.25	0.410
Total cholesterol (mmol/L)	4.34 ± 0.48	4.60 ± 0.88	4.04 ± 0.68	0.104
LDL-cholesterol (mmol/L)	2.24 ± 0.40	2.60 ± 0.69	2.23 ± 0.52	0.141
HDL-cholesterol (mmol/L)	1.73 ± 0.24	1.69 ± 0.40	1.53 ± 0.32	**0.016**
Urinary cortisol (nmol/L)	574 ± 270	497 ± 155	466 ± 164	0.604
**Dietary intake**				
Energy (kcal/day)	2756 ± 603	2705 ± 602	2656 ± 485	0.835
Carbohydrates (g/day)	258 ± 84.76	241 ± 73.92	251 ± 63.80	0.840
Sugar (g/day)	116 ± 35.98	112 ± 35.04	119 ± 42.24	0.989
Fiber (g/day)	45.02 ± 23.03	42.12 ± 14.65	40.76 ± 15.17	0.915
Protein (g/day)	103 ± 30.00	110 ± 31.86	109 ± 29.64	0.577
Total fat (g/day)	143 ± 28.01	142 ± 35.35	134 ± 28.61	0.249
SFAs (g/day)	37.67 ± 9.56	38.18 ± 11.04	38.11 ± 12.96	0.825
MUFAs (g/day)	69.35 ± 15.10	69.06 ± 17.17	62.13 ± 15.24	0.326
PUFAs (g/day)	25.70 ± 7.10	23.99 ± 7.25	23.37 ± 7.25	0.617
**Polyphenol intake**				
Flavonoids (mg/day)	659 ± 392	855 ± 676	664 ± 620	0.463
Phenolic acids (mg/day)	178 ± 113	208 ± 155	169 ± 79.87	0.978
Stilbenes (mg/day)	0.28 ± 0.44	0.31 ± 0.45	0.11 ± 0.13	0.067
Lignans (mg/day)	5.69 ± 7.03	4.76 ± 5.22	4.03 ± 4.06	0.716
**Present in peanuts**				
*m*-coumaric acid (mg/day)	0.79 ± 0.76	0.64 ± 0.73	0.29 ± 0.34	**0.035**
*o*-coumaric acid (mg/day)	0.49 ± 0.39	0.39 ± 0.38	0.23 ± 0.20	0.052
*p*-coumaric acid (mg/day)	0.94 ± 0.43	0.72 ± 0.36	0.70 ± 0.38	0.144
Resveratrol (mg/day)	0.05 ± 0.03	0.04± 0.02	0.03 ± 0.02	0.668

Data are expressed as mean ± SD. SRP: skin-roasted peanuts; PB: peanut butter; CB: control butter; MET/week: metabolic equivalent of task-minutes per week. BMI: body mass index; SFAs: saturated fatty acids; MUFAs: monounsaturated fatty acids; PUFAs: polyunsaturated fatty acids. *p*-value column refers to differences between groups at baseline. *p*-values < 0.05 are statistically significant and were calculated by chi-square test for categorical variables and Kruskal–Wallis test for continuous variables.

**Table 2:** Effect of intervention on telomere length after 6 months of intervention.

		SRP vs. CB		PB vs. CB	
	Models	Difference Time-Exposure (95% CI)	*p*-Value	Difference Time-Exposure (95% CI)	*p*-Value
Telomere length	Model 1	0.50 (−0.02, 1.02)	0.058	0.12 (−0.40, 0.64)	0.653
	Model 2	0.53 (0.01, 1.05)	**0.048**	0.12 (−0.42, 0.66)	0.661

SRP: skin-roasted peanuts; PB: peanut butter; CB: control butter. Generalized estimating equation (GEE) models were used to estimate the effect (difference) of intervention among study groups. Model 1: adjusted by sex and age; Model 2: adjusted as in Model 1 plus BMI, physical activity, and energy intake. *p*-value: group × time interaction. Values <0.05 are statistically significant and highlighted in bold.

**Table 3:** Association between anthropometric, biochemical, and dietary variables that changed with intervention and Δ telomere length.

			Telomere Length	
		*n*	B (CI)	*p*-Value
Waist-to-hip Ratio	Model 3	58	−0.17 (−0.72, 0.38)	0.534
HDL-cholesterol	Model 3	57	0.23 (−0.68, 1.13)	0.615
Carbohydrates intake	Model 3	58	−0.03 (−0.46, 0.39)	0.880
Fiber intake	Model 3	58	−0.08 (−0.65, 0.49)	0.782
MUFA intake	Model 3	58	0.35 (0.01, 0.69)	**0.046**
Vitamin E intake	Model 3	58	0.09 (−0.39, 0.56)	0.714
*m*-Coumaric acid	Model 3	58	0.42 (0.18, 0.67)	**0.012**
*o*-Coumaric acid	Model 3	58	0.24 (−0.32, 0.81)	0.394
*p*-Coumaric acid	Model 3	58	−0.35 (−1.79, 1.09)	0.626
Resveratrol	Model 3	58	0.68 (−0.70, 2.05)	0.082
Flavones	Model 3	58	0.31 (−0.33, 0.94)	0.333
Anthocyanins	Model 3	58	0.46 (−0.30, 1.23)	0.225

MUFA: monounsaturated fatty acids. B: non-standardized coefficient. CI: confidence interval. Model 3: fully adjusted model by sex, age, body mass index, physical activity, energy intake, and telomere length at baseline. Models 1 and 2 are shown in Table A3. *p*-values shown in bold are statistically significant *p* < 0.050.

## Data Availability

Data described in the manuscript, code book, and analytic code will be made available upon request.

## Appendix A

**Table A1:** Intervention effect on anthropometric, biochemical, and clinical parameters at 6 months of intervention.

	Models	SRP vs. CB	*p*-Value	PB vs. CB	*p*-Value
		Mean Difference 6 Moth (95% CI)		Mean Difference 6 Moth (95% CI)	
**Anthropometric measurements**					
Weight (Kg)	Model 1	−0.01 (−2.44, 2.42)	0.991	−0.69 (−2.94, 1.56)	0.547
	Model 2	0.09 (−0.49, 0.68)	0.752	−0.24 (−0.73, 0.24)	0.328
BMI (kg/m^2^)	Model 1	−0.03 (−0.86, 0.79)	0.935	−0.16 (−0.91, 0.58)	0.670
	Model 2	−0.03 (−0.86, 0.79)	0.939	−0.20 (−0.95, 0.55)	0.602
Waist circumference (cm)	Model 1	−0.12 (−2.03, 1.79)	0.902	−0.14 (−2.36, 2.09)	0.904
	Model 2	−0.16 (−1.35, 1.04)	0.797	0.19 (−1.72, 2.09)	0.849
Waist-to-hip ratio	Model 1	−0.03 (−0.07, 0.01)	0.076	−0.03 (−0.06, 0.01)	0.086
	Model 2	−0.03 (−0.05, −0.01)	**0.027**	−0.02 (−0.04, 0.01)	0.139
Body fat (%)	Model 1	−0.00 (−1.74, 1.74)	0.999	−0.34 (−2.10, 1.41)	0.702
	Model 2	0.07 (−0.90, 1.05)	0.880	−0.11 (−0.99, 0.77)	0.811
Visceral fat	Model 1	−0.13 (−0.96, 0.70)	0.762	−0.11 (−0.86, 0.63)	0.766
	Model 2	−0.07 (−0.44, 0.30)	0.707	−0.03 (−0.39, 0.34)	0.878
Muscle mass (%)	Model 1	0.37 (−0.73, 1.47)	0.511	0.24 (−0.78,1.272)	0.642
	Model 2	0.39 (−0.57, 1.33)	0.436	0.15 (−0.60, 0.90)	0.695
**Blood pressure**					
Systolic blood pressure (mmHg)	Model 1	0.74 (−7.86, 9.33)	0.867	−3.13 (−10.66, 4.39)	0.415
	Model 2	0.80 (−7.19, 8.80)	0.844	−3.10 (−10.11, 3.90)	0.385
Diastolic blood pressure (mmHg)	Model 1	2.60 (−4.61, 9.81)	0.480	0.78 (−5.38, 6.93)	0.805
	Model 2	2.64 (−4.20, 9.48)	0.449	1.26 (−4.74, 7.25)	0.681
**Biochemical parameters**					
Glucose (mmol/L)	Model 1	0.07 (−0.12, 0.27)	0.456	−0.05 (−0.23, 0.14)	0.628
	Model 2	0.08 (−0.11, 0.27)	0.387	−0.03 (−0.21, 0.16)	0.791
Triglyceride (mmol/L)	Model 1	0.08 (−0.13, 0.30)	0.444	−0.01 (−0.23, 0.21)	0.938
	Model 2	0.09 (−0.13, 0.31)	0.435	0.02 (−0.19, 0.23)	0.857
Total cholesterol (mmol/L)	Model 1	−0.06 (−0.73, 0.60)	0.848	−0.15 (−0.84, 0.54)	0.676
	Model 2	−0.05 (−0.72, 0.62)	0.883	−0.13 (−0.83, 0.57)	0.717
HDL-cholesterol (mmol/L)	Model 1	0.20 (0.01, 0.39)	**0.035**	0.16 (−0.06, 0.38)	0.162
	Model 2	0.20 (0.02, 0.37)	**0.030**	0.14 (−0.06, 0.34)	0.168
LDL-cholesterol (mmol/L)	Model 1	−0.07 (−0.55, 0.42)	0.783	−0.08 (−0.63, 0.47)	0.771
	Model 2	−0.04 (−0.53, 0.44)	0.859	−0.04 (−0.59, 0.50)	0.877

SRP: skin-roasted peanuts; PB: peanut butter; CB: control butter; BMI: body mass index. Generalized estimating equation (GEE) models were used to estimate effect (difference) of intervention among study groups. Model 1: adjusted by sex and age; Model 2: adjusted as in Model 1 plus BMI, physical activity, and energy intake. *p*-value: group × time interaction. Values < 0.05 are statistically significant and highlighted in bold.

**Table A2:** Dietary intake after 6 months of intervention.

Dietary Intake	Models	SRP vs. CB	*p*-Value	PB vs. CB	*p*-Value
		Difference Time- Exposure (95% CI)		Difference Time-Exposure (95% CI)	
**Nutrient intake**					
Carbohydrates (g/day)	Model 1	0.85 (−25.80, 27.49)	0.950	2.85 (−24.73, 30.43)	0.840
	Model 2	9.56 (−4.49, 23.63)	0.182	7.79 (−7.99, 23.58)	0.334
Sugars (g/day)	Model 1	7.95 (−6.13, 22.04)	0.269	5.45 (−8.01, 18.92)	0.427
	Model 2	11.59 (0.04, 23.15)	**0.049**	6.42 (−3.66, 16.50)	0.212
Fiber (g/day)	Model 1	3.38 (−1.17, 7.94)	0.146	2.78 (−2.13, 7.70)	0.267
	Model 2	4.88 (1.77, 7.99)	**0.002**	3.65 (0.45, 6.85)	**0.025**
Protein (g/day)	Model 1	−4.57 (−13.69, 4.54)	0.325	−5.25 (−14.96, 4.47)	0.290
	Model 2	−0.78 (−7.94, 6.39)	0.832	−3.56 (−10.47, 3.35)	0.313
Total fat (g/day)	Model 1	11.12 (−1.24, 23.48)	0.078	−5.97 (−19.18, 7.23)	0.375
	Model 2	6.27 (−0.10, 12.63)	0.054	−3.13 (−10.21, 3.95)	0.386
Saturated fat (g/day)	Model 1	−0.87 (−4.86, 3.12)	0.670	−1.50 (−5.66, 2.67)	0.481
	Model 2	0.60 (−1.77, 2.96)	0.622	−0.71 (−2.85, 1.43)	0.515
Monounsaturated fat (g/day)	Model 1	7.41 (0.79, 14.02)	**0.028**	−5.41 (−12.36, 1.54)	0.127
	Model 2	5.29 (0.12, 10.71)	0.056	−4.37 8 (10.71, 0.12)	0.139
Polyunsaturated fat (g/day)	Model 1	0.60 (−3.11, 4.31)	0.752	1.59 (−2.22, 5.40)	0.413
	Model 2	1.34 (−1.31, 4.00)	0.322	2.28 (−0.57, 5.13)	0.117
Energy, kcal/d	Model 1	−92.80 (−299, 114)	0.379	−52.09 (−269, 165)	0.638
	Model 2				
**Food consumption**					
Fruits (g/day)	Model 1	26.23 (−48.62, 101)	0.492	−19.05 (−67.13, 29.03)	0.437
	Model 2	34.04 (−37.49, 106)	0.351	−21.80 (−66.30, 22.70)	0.337
Vegetables (g/day)	Model 1	−20.07 (−50.37, 10.21)	0.194	−9.79 (−44.11, 24.54)	0.576
	Model 2	−14.83 (−45.01, 15.36)	0.336	−8.79 (−42.36, 24.78)	0.608
Cereals (g/day)	Model 1	−20.40 (−57.86, 17.05)	0.286	−22.72 (−60.76)	0.242
	Model 2	−12.90 (−43.74, 17.95)	0.412	−15.70 (−47.94, 17.95)	0.412
Potatoes (g/day)	Model 1	−3.25 (−27.33, 20.82)	0.791	10.30 (−12.33, 33.53)	0.385
	Model 2	−2.43 (−26.79, 21.93)	0.845	12.28 (−12.24, 36.80)	0.326
Legumes (g/day)	Model 1	4.28 (−8.27, 16.83)	0.504	−0.77 (−11.87, 10.34)	0.892
	Model 2	5.02 (−7.10, 17.16)	0.417	−0.48 (−11.50, 10.54)	0.932
Dairy products (g/day)	Model 1	45.74 (−38.52, 130)	0.287	25.21 (−63.53, 113)	0.578
	Model 2	51.76 (−30.19, 134)	0.216	31.07 (−57.34, 119)	0.491
Eggs (g/day)	Model 1	7.68 (−4.45, 19.81)	0.214	−12.11 (−38.11, 13.89)	0.361
	Model 2	10.09 (−2.60, 22.77)	0.119	−11.25 (−35.84, 13.35)	0.370
Fish and sea food (g/day)	Model 1	−11.02 (−24.52, 2.48)	0.109	−0.76 (−12.30, 10.78)	0.897
	Model 2	−7.89 (−22.90, 7.12)	0.303	0.21 (13.41, 13.83)	0.976
Meat/meat products (g/day)	Model 1	−0.39 (−20.89, 20.11)	0.970	−18.35 (−39.39, 2.68)	0.087
	Model 2	3.05 (−18.52, 24.62)	0.782	−18.49 (−38.76, 1.78)	0.074
Oils and butter (g/day)	Model 1	8.42 (−7.76, 24.59)	0.308	1.23 (−10.36, 12.83)	0.834
	Model 2	10.28 (−6.51, 27.06)	0.230	2.34 (−8.51, 13.21)	0.672
EVOO (g/day)	Model 1	0.82 (−6.99, 8.62)	0.838	−6.15 (−13.16, 0.86)	0.086
	Model 2	1.23 (−6.97, 9.43)	0.769	−6.08 (−13.49, 1.33)	0.108
Pastries (g/day)	Model 1	0.32 (−15.83, 16.47)	0.969	3.14 (−12.53, 18.81)	0.694
	Model 2	3.28 (−10.53, 17.10)	0.641	5.51 (−6.15, 17.17)	0.354
**Micronutrients**					
B-group vitamins	Model 1	10.06 (−41.38, 61.49)	0.702	2.39 (−51.75, 56.52)	0.931
	Model 2	26.51 (−15.94, 68.97)	0.221	11.62 (−31.53, 54.76)	0.598
Vitamin A	Model 1	67.44 (−195, 330)	0.614	−28.12 (−399, 344)	0.882
	Model 2	129 (−129, 386)	0.327	−9.07 (−369, 351)	0.961
Vitamin D	Model 1	0.25 (−0.59, 1.09)	0.554	0.31 (−0.66, 1.28)	0.533
	Model 2	0.50 (−0.49, 1.49)	0.329	0.35 (−0.66, 1.37)	0.499
Vitamin E	Model 1	3.38 (0.96, 5.80)	**0.006**	3.97 (1.10, 6.85)	**0.007**
	Model 2	2.76 (0.85, 4.67)	**0.005**	3.56 (1.25, 5.84)	**0.002**
Vitamin C	Model 1	−16.78 (−45.88, 12.36)	0.259	−10.17 (−40.49, 20.15)	0.511
	Model 2	−12.33 (−42.59, 17.93)	0.425	−11.47 (−42.59, 19.65)	0.470
Sodium	Model 1	−45.05 (−395, 305)	0.801	−135 (−475, 203)	0.432
	Model 2	59.28 (−257, 375)	0.713	−88 (−353, 176)	0.512
Magnesium	Model 1	−6.43 (−64.39, 51.53)	0.828	−15.74 (−74.86, 43.38)	0.602
	Model 2	14.59 (−15.89, 45.07)	0.348	−1.38 (−31.28, 28.52)	0.928
Potassium	Model 1	−15.41 (−192, 162)	0.865	−68.76 (−243, 106)	0.440
	Model 2	51.18 (−68.50, 171)	0.402	−30.80 (−147, 85.22)	0.603
Iron	Model 1	−0.02 (−1.56, 1.52)	0.981	−0.53 (−2.25, 1.19)	0.546
	Model 2	0.61 (−0.39, 1.60)	0.230	−0.16 (−1.14, 0.82)	0.748
Zinc	Model 1	0.05 (−1.09, 1.19)	0.933	−1.02 (−2.36, 0.32)	0.136
	Model 2	0.54 (−0.22, 1.29)	0.165	−0.75 (−1.51, 0.01)	0.053
**Polyphenols**					
Flavanols	Model 1	89.34 (−218, 397)	0.570	−122 (−493, 250)	0.521
	Model 2	113 (−182, 409)	0.451	−113 (−460, 234)	0.523
Flavonols	Model 1	−3.94 (−11.54, 3.66)	0.310	0.50 (−5.37, 6.37)	0.868
	Model 2	−2.57 (−9.25, 4.10)	0.450	1.57 (−3.55, 6.68)	0.548
Flavanones	Model 1	−12.73 (−34.81, 9.35)	0.258	−3.34 (−19.88, 13.21)	0.692
	Model 2	−12.27 (−20.90, 13.39)	0.274	−3.76 (−20.90, 13.39)	0.667
Flavones	Model 1	−23.68 (−46.04, −1.33)	**0.038**	−24.84 (−48.34, −1.34)	**0.038**
	Model 2	−18.72 (−41.08, 3.65)	0.101	−20.41 (−41.66, 0.83)	0.060
Isoflavonoids	Model 1	−2.74 (−8.65, 3.16)	0.362	−2.49 (−5.56, 0.58)	0.112
	Model 2	−2.49 (−8.03, 3.05)	0.378	−2.11 (−4.83, 0.62)	0.130
Anthocyanins	Model 1	−6.48 (−12.46, −0.50)	**0.034**	−2.18 (−7.58, 3.22)	0.429
	Model 2	−5.71 (−11.43, 0.01)	0.051	−2.01 (−7.43, 3.42)	0.468
Phenolic acids	Model 1	18.75 (−91.99, 129)	0.740	18.86 (−100, 138)	0.757
	Model 2	42.41 (−57.45, 142)	0.405	30.13 (−67.03, 127)	0.543
Stilbenes	Model 1	0.09 (−0.01, 0.28)	0.351	0.10 (−0.07, 0.21)	0.265
	Model 2	−0.08 (−0.27, 0.11)	0.424	−0.09 (−0.27, 0.08)	0.272
Lignans	Model 1	−0.19 (−2.90, 2.53)	0.892	0.23 (−2.96, 3.43)	0.885
	Model 2	−0.10 (−2.69, 2.49)	0.938	0.45 (−2.60, 3.50)	0.771
**Present in peanuts**					
*m*-coumaric acid	Model 1	0.40 (0.18, 0.63)	**<0.001**	0.41 (0.25, 0.57)	**<0.001**
	Model 2	0.43 (0.21, 0.66)	**<0.001**	0.41 (0.25, 0.57)	**<0.001**
*o*-coumaric acid	Model 1	1.43 (0.81, 2.04)	**<0.001**	0.04 (−0.05, 0.14)	0.376
	Model 2	1.45 (0.85, 2.04)	**<0.001**	0.03 (−0.08, 0.13)	0.631
*p*-coumaric acid	Model 1	6.15 (5.95, 6.36)	**<0.001**	12.99 (12.42, 13.56)	**<0.001**
	Model 2	6.19 (6.02, 6.37)	**<0.001**	13.01 (12.43, 13.59)	**<0.001**
Resveratrol	Model 1	0.07 (0.05,0.08)	**<0.001**	0.08 (0.06, 0.09)	**<0.001**
	Model 2	0.07 (0.05, 0.09)	**<0.001**	0.08 (0.07, 0.09)	**<0.001**

SRP: skin-roasted peanuts; PB: peanut butter; CB: control butter; EVOO: extra virgin olive oil. Generalized estimating equation (GEE) models were used to estimate effect (difference) of intervention among study groups. Model 1: adjusted by sex and age; Model 2: adjusted as in Model 1 plus BMI, physical activity, and energy intake. *p*-value: group × time interaction. Values < 0.05 are statistically significant and highlighted in bold.

**Table A3:** Association between anthropometric, biochemical, and dietary variables that changed with intervention and Δ telomere length.

		*n*	Telomere Length	
			B (CI)	*p*-Value
Waist-to-hip ratio	Model 1	58	−0.11 (−0.73;0.52)	0.731
	Model 2	58	−0.08 (−0.66, 0.50)	0.791
HDL-cholesterol	Model 1	57	0.22 (−0.82; 1.32)	0.643
	Model 2	57	0.03 (−0.94, 1.00)	0.947
Carbohydrates intake	Model 1	58	0.08 (−0.66, 0.83)	0.822
	Model 2	58	0.15 (−0.55, 0.85)	0.660
Fiber intake	Model 1	58	−0.01 (−0.78, 0.77)	0.990
	Model 2	58	0.07 (−0.66,0.81)	0.838
MUFA intake	Model 1	58	0.58 (0.11, 1.05)	**0.016**
	Model 2	58	0.46 (0.02, 0.93)	**0.048**
Vitamin E intake	Model 1	58	−0.15 (−0.82, 0.52)	0.663
	Model 2	58	−0.09 (−0.65, 0.48)	0.760
*m*-Coumaric acid	Model 1	58	0.24 (−0.31, 0.79)	0.627
	Model 2	58	0.23 (−0.29, 0.75)	0.630
*o*-Coumaric acid	Model 1	58	−0.25 (−1.27, 0.76)	0.619
	Model 2	58	−0.27 (−1.21, 0.67)	0.571
*p*-Coumaric acid	Model 1	58	−0.06 (−1.90, 1.78)	0.949
	Model 2	58	0.06 (−1.54, 1.67)	0.936
Resveratrol	Model 1	58	1.20 (0.01, 2.39)	**0.048**
	Model 2	58	1.52 (0.37, 2.67)	**0.011**
Flavones	Model 1	58	−0.07 (−0.80, 0.65)	0.838
	Model 2	58	−0.03 (−0.72, 0.66)	0.929
Anthocyanins	Model 1	58	0.79 (0.02, 1.56)	**0.044**
	Model 2	58	0.81 (−0.02, 1.66)	0.057

MUFA: monounsaturated fatty acids. B: Non-standardized coefficient. CI: Confidence interval. Model 1: sex and age. Model 2: adjusted as in model 1 plus body mass index, physical activity, and energy intake. *p*-values shown in bold are statistically significant *p* < 0.050.
